# Clinical Characteristics and Immediate-Outcome of Children Mechanically Ventilated in PICU of Pakistan

**DOI:** 10.12669/pjms.305.5159

**Published:** 2014

**Authors:** Beenish Mukhtar, Naveedur R. Siddiqui, Anwarul Haque

**Affiliations:** 1 Beenish Mukhtar, MBBS, Department of Paediatrics and Child Health, Aga Khan University Hospital, Stadium Road, Karachi 74800, Pakistan.; 2 Naveedur R. Siddiqui, MBBS, FCPS, Department of Paediatrics and Child Health, Aga Khan University Hospital, Stadium Road, Karachi 74800, Pakistan.; 3 Anwarul Haque, MBBS, Department of Paediatrics and Child Health, Aga Khan University Hospital, Stadium Road, Karachi 74800, Pakistan.

**Keywords:** Children, PICU, Mechanical Ventilation, Outcome

## Abstract

***Back ground and Objective: ***Mechanical Ventilation (MV) is frequently used as one of the most frequent life-supportive technology in Pediatric Intensive Care Units (PICUs). Very little data is available from Asian countries like Pakistan regarding use of MV in PICUs. Our objective was to assess the frequency, indications and immediate-outcomes in mechanically ventilated pediatric patients in tertiary-care center of developing country.

***Methods: ***Retrospective cohort study of critically ill pediatric patients admitted in PICU of Aga Khan University Hospital, who required MV for more than 24-hour over two-year period.

***Results: ***A total of 605 patients were admitted to PICU, 307 (50.7%) patients required MV support for >24hr. The median age was 3 years (IQR 6 month to 6 yr 2 months), and male was 59.6% (183/307). Common indications for MV was neurological illness 35.8%, followed by respiratory diseases in 20.8% patients and cardiac diseases in 13%; and 30.3 % patients were ventilated for other reasons. The median length of MV was 2.1 days. 9.4% developed complications and atelectasis (4.6%) was the most common. The mortality rate of children mechanically ventilated was 30.3% as compared to the overall mortality rate of in PICU was 16.3%. The long duration (> 10 days) and cardiogenic shock were identified as independent risk factor associated with increased mortality.

***Conclusion: ***About half of PICU admission required mechanical ventilation for more than 24 hours. The neurological illness was the most common reason for ventilation. The low incidence of complication rate and relatively high mortality in cardiac cases and long duration of mechanical ventilation were noted in our cohort.

## INTRODUCTION

Mechanical Ventilation (MV) is a life-supporting device, invasive technology of intensive care unit, to mimic the respiratory physiological function at the time of either impending or acute respiratory failure.^1^ MV is expensive, labor-intensive and is associated with adverse effects lead to death. With the major advances in the field of mechanical ventilation with introduction of several new modes, its use is becoming simple and easy and is growing very fast in the pediatric intensive care units (PICUs). The percentage of children receiving mechanical ventilation in PICUs ranges from 17 -64% in developed countries where PICUs is a mature and established discipline of medicine.^2^^-^^4^ Very little data is available from Asian countries like Pakistan regarding use of MV in PICUs.^5^^,^^6^

The objective of this study was to assess the frequency of its use, indications, complications and immediate-outcome of children receiving mechanical ventilation in a PICU of a tertiary-care hospital from a developing country.

## METHODS

We retrospectively reviewed the medical records of all children range from one-month to sixteen-years who received mechanical ventilation for more than 24-hour in pediatric intensive care unit from our local database from January 2011 to December 2012.

AKUH is a 600-bedded tertiary-care, university- hospital and four-bed was allocated to PICU with annual census of approximately 600 patients. This PICU is a closed, multidisciplinary, and staffed by one full-time pediatric intensivist, two clinical fellow of pediatric critical care medicine, two senior pediatric residents with one-one nurse: patient ratio. They are responsible for managing patients on ventilator from initiation to liberation from mechanical ventilation. Each bed has mechanical ventilation and equipped with monitor along with end-tidal CO_2 _monitoring. This unit has own ABG machine as well as portable X-ray machine with PACS system. We use Simens Servo 300 and Purriten-Bannete 480 for respiratory support in our PICU. Mechanical ventilation in all patients was initiated through an orotracheal tube.

All children during mechanical ventilation were getting continuous infusion of midazolam and morphine as sedation and analgesia mostly. The neuromuscular blocking agent was used infrequently and was needed only during high ventilatory support for severe hypoxemic respiratory failure, shock to improve the mismatch between demand and supply and during refractory intracranial hypertension, etc. The sedation holiday was done routinely in our unit to prevent drug accumulation.

The most commonly used mode on conventional mechanical ventilation was SIMV-PC/PS with normal respiratory physiological parameters for such age and clinical condition in our unit. The initial parameter was set according to need of patients and adjusted according to clinical variables, chest X-ray and arterial blood gas analysis (ABG) as described by Rotta et al.^7^ Then subsequent parameters on mechanical ventilation were modified according to need of oxygenation and ventilation through SpO_2_ and end-tidal CO_2_ monitoring or blood gas analysis. We infrequently use ABG and Chest X-ray for ventilatory management in our unit. The suctioning practices of endotracheal tube and saline nebulizer were used by recommendations. Efforts were made to ventilate effectively with minimal setting. All children were monitored for complications. All patients were liberated from MV when clinical condition has improved and after passing spontaneous breathing trial. All patients were monitored for signs of clinical deterioration after extubation for twenty-four in intensive care unit.

Data was collected on structured propforma included the basic demographic profile (age, gender, primary diagnosis), reasoning for MV, length of MV and PICU stay, complications of MV and outcome of patient (either as discharge alive or expired). Frequency and mean ± Standard deviation (SD) or median with IQR were computed using SPSS 20 for respective variables. All critically ill patients who expired were analyzed under Logistic Regression Analysis and Variables with p value < 0.1 were undergone multivariate analysis with p value <0.05 with OR 95% confidence interval CI to find out the associated risk factor with mortality among the critically ill patients who underwent mechanical ventilation. This study was approved by the local ethical committee.

## RESULTS

During two-year study period, 605 patients were admitted in our PICU and 307 (50.7%) of them received mechanical ventilation for more than 24 hour. The median age of ventilated patients were 3 years (IQR 6 months to 74 months), and most of them were male 59.6% (183/307). The age was further divided in to three subcategories: <12 month (n=99), 1-5 yr (n=98) and >5 yr (n=110).

**Fig.1:**
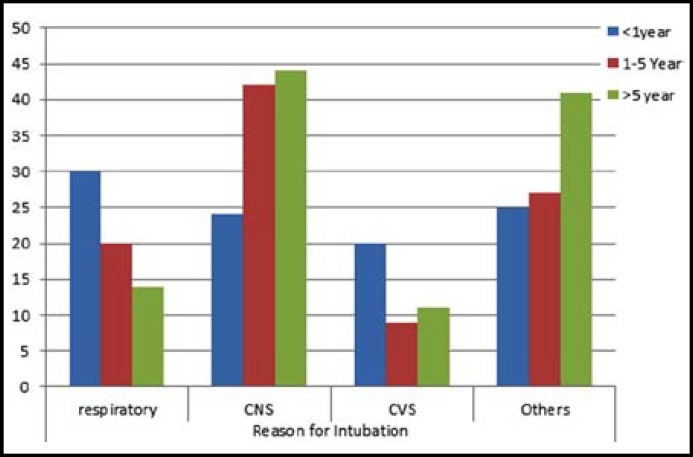
Indications of MV in different age groups

The indications for mechanical ventilation in PICU were divided into four major categories including acute neurological illness (35.8%), respiratory illness (20.8%), cardiac failure (13%) and miscellaneous group (30.3%) mostly involve safety of airway like postoperative patients and septic shock, etc. Further sub categorization is shown in Table-I. Fig.1 revealed the different indications of MV in different age groups. The median mechanically ventilated days was 2.1 days (IQR 0.9-5.4days). The complication rate in MV children was 9.4% (29/307). These included lobar atelectasis 4.6% (14/307), ventilator associated pneumonia (3.3%), pulmonary hemorrhage (1.3%) and pneumothorax (0.3%).

The mortality rate among the critically ill mechanically ventilated patients in our cohort was of 30.5% (93/307) as compared to the overall mortality rate of critically ill patients in the PICU of was 16.3% (99/605). Univariate and multiple regression analysis were done on mechanically ventilated patients in PICU. We found that longer duration mechanical ventilation (>10days) and cardiogenic shock as an independent predictors of mortality in mechanically children in PICU.

## DISCUSSION

We found that 50.7% (307/605) of infant and children admitted to our PICU received MV for more than 24 hour. The percentage of pediatric patients mechanically ventilated in different PICUs varied from 14 -60%.^2^^-^^4^^,^^8^^,^^9^ Vigayakumary et al. reported that 52% of children received MV in PICU of Sri Lanka.^6^ Wolfler et al. reported that 34.6% of PICU admission in Italy required MV for >24 hrs.^3^ Khemani et al. published that 30% of children in a cross-section of United States PICUs were mechanically ventilated.^9^

**Table-I:** Indications of MV and sub-categories

***Disease***	***N=number (%)***
Respiratory	64 (20.8)
Pneumonia	47 (15.3)
Bronchiolitis	6 (2.0)
Upper airway Obstruction	4 (1.3)
Hemorrhage	4 (1.3)
Muscular disease	3 (1.0)
CNS	110 (35.8)
Meningitis/Encephalitis	37 (12.1)
Traumatic Coma	18 (5.9)
Nontraumatic Coma	33 (10.7)
HIE	9 (2.9)
Neuromuscular Disease	14 (4.6)
Cardiovascular	40 (13)
CCF	19 (6.2)
Shock	22 (7.2)
Miscellaneous	93 (30.3)
Postoperative	54 (17.6)
Secure Airway	38 (12.4)

The most common indication of MV was acute neurological illnesses (35.8%) in our study. Like us, Wolfler et al. found neurological illness was the most common reason of MV in PICUs.^3^ However, several other reports that respiratory failure due to respiratory illness was the most common indication of MV in PICUs.^2^^,^^10^^,^^11^ The most likely explanation for this change in pattern was the increased use of non-invasive ventilation through high-flow nasal cannula and Bi-PAP in the early phases of acute respiratory illness like bronchiolitis and pneumonia as initial mode of respiratory support.

The SIMV was the most commonly used as an initial mode of ventilatory support because of our comfort and confidence. Several published reports also found that SIMV was commonly used as initial mode on MV.^10^^,^^11^ However, there has been increasing use of volume target ventilation in PICUs.

The complications are common among mechanically ventilated children in PICU.^12^ The complication rate in our cohort was 9.2% as compared to 42.8% reported by Kendirili et al. Like Kendirili et al., the most common complication was atelectasis.^10^

The duration of mechanical ventilation was 4-6 days in few published reports.^2^^,^^3^ The average length of mechanical ventilation in our PICU was 2.1 days. Only 6.2% (19) required ventilatory support for more than 10 days.

This study also tried to find out the predictors of mortality in mechanically ventilated children in PICU. Acute cardiac failure (p <0.001) and prolonged mechanical ventilation (>10days) (p <0.05) were significantly important predictor of mortality in mechanically ventilated children in our PICU.

The mortality rate of our ventilated children was 30.5%. The mortality rates of children mechanically ventilated among these reports were different.^3^^,^^4^^,^^10^ Shaukat et al. and Kendiril et al. reported the survival rate were 63% and 58.3% from Pakistan and Turkey respectively in the past.^5^^,^^10^ Vigayakumary et al. reported 27.6% mortality rate among mechanically ventilated patients which is close to our result.^6^ In developed countries, the overall mortality rates in mechanically ventilated patients in PICUs were < 2%.^4^ There are several reasons for this major difference in the mortality rate of MV children. Several advantages including higher number of postoperative cases in their PICUs, trained staff, availability of respiratory therapist for ventilatory management, early presentation of illness are known for established PICUs in developed countries. We have several disadvantages including lack of respiratory therapist services, lack of education and training of MV as well as delayed presentation with multiorgan dysfunction syndrome. To improve the outcome of MV children in PICUs, we need effective, organized and structured educational courses from basic concept to clinical application for all physicians and nurses involved in the care of critically ill children receiving mechanical ventilation. As we gain experience in the ventilation our complications rate and mortality related to mechanical ventilation would also decrease.

## Authors Contribution:

**BM:** Manuscript writing and data collection. **NRS:** Statistical analysis.

**AH:** Conception, design and guarantor of manuscript.

## Notes

***Financial Disclosure: ***None.
